# Association of *ABI3* and *PLCG2* missense variants with disease risk and neuropathology in Lewy body disease and progressive supranuclear palsy

**DOI:** 10.1186/s40478-020-01050-0

**Published:** 2020-10-22

**Authors:** Samantha L. Strickland, Hélène Morel, Christian Prusinski, Mariet Allen, Tulsi A. Patel, Minerva M. Carrasquillo, Olivia J. Conway, Sarah J. Lincoln, Joseph S. Reddy, Thuy Nguyen, Kimberly G. Malphrus, Alexandra I. Soto, Ronald L. Walton, Julia E. Crook, Melissa E. Murray, Bradley F. Boeve, Ronald C. Petersen, John A. Lucas, Tanis J. Ferman, Ryan J. Uitti, Zbigniew K. Wszolek, Owen A. Ross, Neill R. Graff-Radford, Dennis W. Dickson, Nilüfer Ertekin-Taner

**Affiliations:** 1 Department of Neuroscience, Mayo Clinic Florida, Jacksonville, FL 32224 USA; 2 Department of Health Sciences Research, Mayo Clinic Florida, Jacksonville, FL 32224 USA; 3 Department of Neurology, Mayo Clinic Minnesota, Rochester, MN 55905 USA; 4 Department of Psychiatry and Psychology, Mayo Clinic Florida, Jacksonville, FL 32224 USA; 5 Department of Neurology, Mayo Clinic Florida, Jacksonville, FL 32224 USA

**Keywords:** Lewy body disease, Dementia with Lewy bodies, Progressive supranuclear palsy, Neuropathology, Tau, Amyloid ß, Genetic associations

## Abstract

Missense variants *ABI3*_rs616338-T and *PLCG2*_rs72824905-G were previously associated with elevated or reduced risk of Alzheimer’s disease (AD), respectively. Despite reports of associations with other neurodegenerative diseases, there are few studies of these variants in purely neuropathologically diagnosed cohorts. Further, the effect of these mutations on neurodegenerative disease pathologies is unknown. In this study, we tested the effects of *ABI3*_rs616338-T and *PLCG2*_rs72824905-G on disease risk in autopsy cohorts comprised of 973 patients diagnosed neuropathologically with Lewy body disease (LBD-NP) and 1040 with progressive supranuclear palsy (PSP), compared to 3351 controls. LBD-NP patients were further categorized as high, intermediate and low likelihood of clinical dementia with Lewy bodies (DLB-CL) based on DLB Consortium criteria. We also tested for association with both Braak neurofibrillary tau tangle (n_Total_ = 2008, n_PSP_ = 1037, n_LBD-NP_ = 971) and Thal phase amyloid plaque scores (n_Total_ = 1786, n_PSP_ = 1018, n_LBD-NP_ = 768). Additionally, 841 PSP patients had quantitative tau neuropathology measures that were assessed for genetic associations. There was no statistically significant association with disease risk for either LBD-NP or PSP in our study. LBD intermediate category disease risk was significantly associated with *ABI3*_rs616338-T (OR = 2.65, 95% CI 1.46–4.83, *p* = 0.001). *PLCG2*_rs72824905-G was associated with lower Braak stage (ß = − 0.822, 95% CI − 1.439 to − 0.204, *p* = 0.009). This effect was more pronounced in the PSP (ß = − 0.995, 95% CI − 1.773 to − 0.218, *p* = 0.012) than LBD-NP patients (ß = − 0.292, 95% CI − 1.283 to 0.698, *p* = 0.563). *PLCG2*_rs72824905-G also showed association with reduced quantitative tau pathology for each lesion type and overall tau burden in PSP (ß = − 0.638, 95% CI − 1.139 to − 0.136, *p* = 0.013). These findings support a role for *PLCG2*_rs72824905-G in suppressing tau neuropathology. *ABI3*_rs616338-T may influence disease risk specifically in the LBD-NP intermediate category comprised of patients with diffuse neocortical or limbic LB, concurrently with moderate or high AD neuropathology, respectively. Our study provides a potential mechanism of action for the missense *PLCG2* variant and suggests a differential disease risk effect for *ABI3* in a distinct LBD-NP neuropathologic category.

**Electronic supplementary material:**

The online version of this article (10.1186/s40478-020-01050-0) contains supplementary material, which is available to authorized users.

## Introduction

Rare, missense variants *ABI3*_rs616338-T and *PLCG2*_rs72824905-G were associated with respectively higher (OR = 1.43) and lower (OR = 0.68) risk of Alzheimer’s disease (AD) in a large study comprised of 48,402 cases and 37,022 controls [22]. In a follow-up study [8], our group validated these associations in AD (n_AD_ = 2743, n_Control_ = 3351). We also evaluated these variants in four additional neurodegenerative diseases including dementia with Lewy bodies (DLB), progressive supranuclear palsy (PSP), Parkinson’s disease (PD) and multiple system atrophy (MSA). In our published DLB cohort, which had autopsy-confirmation for only 22% of 306 patients, both variants had suggestive associations in a direction consistent with AD. In this study, *PLCG2*_rs72824905-G had suggestive associations with increased risk of neuropathologically diagnosed PSP (n_PSP_ = 231) and MSA (n_MSA_ = 128), which is opposite to the direction of effect for this variant in AD.

A subsequent multi-center study by van der Lee et al. [24], which also included our prior results [8], focused only on *PLCG2*_rs72824905-G in multiple neurodegenerative diseases including AD, DLB and PSP. In addition to replicating the association with reduced AD risk, this study also found association between *PLCG2*_rs72824905-G and reduced risk of DLB, but no association with PSP. This multi-center study assessed 1446 DLB patients, of whom only 164 (11%) had neuropathologic diagnosis. Likewise, of the 882 PSP patients in this study, only 260 (29%) were autopsy-confirmed.

Although these two studies collectively support a role for *ABI3*_rs616338-T and *PLCG2*_rs72824905-G in DLB, with a similar effect as in AD, these findings were obtained in DLB cohorts that had 11–22% neuropathologic diagnosis. The results for PSP and *PLCG2*_rs72824905-G association in the two studies were contradictory, but each study had its shortcomings for this diagnosis. Our study [8] had modest size for PSP and the van der Lee et al. [24] PSP cohort was predominantly clinically diagnosed.

The accuracy of clinical diagnosis for DLB is estimated to be 80% based on a recent meta-analysis that utilized neuropathology as the gold standard [21], where the most frequent mis-diagnosis was AD for both false negative (DLB neuropathology) and false positive (not DLB pathology) results. Likewise, in a review of medical records and neuropathologic diagnoses from the CurePSP Brain Bank, 24% of clinically diagnosed PSP patients did not meet neuropathologic criteria of PSP [14]. Most of these misdiagnoses were due to corticobasal degeneration (CBD), MSA and diffuse Lewy body disease. Hence, evaluation of neuropathologically diagnosed cohorts is critical for an accurate assessment of genetic variants in disease risk. Hereafter, we refer to clinically diagnosed DLB patients as DLB-CL and those with neuropathologic diagnosis as Lewy Body Dementia (LBD-NP).

In this study, we addressed this knowledge gap by testing the association of *ABI3*_rs616338-T and *PLCG2*_rs72824905-G in purely autopsy-confirmed cohorts of 973 patients with Lewy body disease (LBD-NP) and 1040 with PSP, compared to 3351 controls. Further, we evaluated the contribution of concurrent AD pathology in LBD-NP [5, 17] to these genetic associations by individually assessing the pathologic categories of LBD-NP patients, determined according to the 2017 DLB Consortium criteria [17]. Finally, we leveraged the existing neuropathologic endophenotypes in these cohorts to determine the role of *ABI3*_rs616338-T and *PLCG2*_rs72824905-G in tau and amyloid ß (Aß) pathologies. Our study represents one of the largest investigations to date of these missense variants for disease risk and neuropathology in autopsy-confirmed LBD-NP and PSP patients. Our findings provide insights for the potential mechanism of action of *ABI3* and *PLCG2* variants and their contribution to LBD-NP and PSP.

## Methods

### Study Populations

We evaluated a total of 5364 participants in this study comprised of 973 LBD-NP and 1040 PSP patients diagnosed with neuropathology, in addition to 3351 controls. We use the term LBD-NP to define our pathologically diagnosed Lewy body disease cases, whereas we use the term DLB-CL to refer to clinically diagnosed cases. All individuals self-reported as Caucasian according to the medical records. Table 1 depicts the demographics of these participants. Pathologic diagnosis of LBD-NP [17] or PSP [12] were made according to published criteria by a single neuropathologist (DWD). Control participants were either cognitively normal at last clinical evaluation (n = 3337) or were autopsy controls who had a Braak score of 2.5 or below and without a neuropathological diagnosis of neurodegenerative disease (n = 14), as previously described [8]. Autopsy-confirmed LBD-NP patients were further categorized as high (n = 423), intermediate (n = 287) or low (n = 263) likelihood for typical clinical DLB-CL based on the 2017 guidelines established by the DLB consortium [17]. According to these criteria, autopsied LBD-NP patients with diffuse neocortical Lewy-related pathology (LRP) and Braak score < 5 or those with limbic (transitional) LRP and Braak < 3 are categorized as high likelihood clinical DLB-CL. Those with diffuse neocortical LRP and Braak > 4 or limbic LRP and Braak 3–4 are intermediate likelihood clinical DLB-CL. All other LRP (i.e. brainstem-predominant, amygdala-predominant, olfactory bulb only) and Braak score (0–6) combinations are low likelihood clinical DLB-CL. This low likelihood category also includes limbic LRP and Braak > 4.

**Table 1:** Study group demographics

Group	Study population	Total N	Mean age ± SD	Female	
				N	%
LBD-NP	Low	263	80.84 ± 11.13	128	48.67
	Intermediate	287	79.63 ± 10.33	163	56.79
	High	423	78.04 ± 7.92	268	63.36
	Combined LBD-NP	973	79.27 ± 9.66	559	57.45
PSP	PSP series 1	230	74.8 ± 7.06	105	45.65
	PSP series 2	810	75.47 ± 7.45	376	46.42
	Combined PSP	1040	75.32 ± 7.37	481	46.25
Controls		3351	80.6 ± 7.1	1844	55.03

All LBD-NP and PSP participants had neuropathologic diagnosis. Controls had either clinical or neuropathologic diagnosis. “Combined LBD-NP” refers to the combined group of LBD-NP patients from all sub-categories. The sub-categories of “High”, “Intermediate”, or “Low” refers to the likelihood of diagnosing typical clinical DLB-CL based on the 2017 DLB-CL Consortium neuropathologic criteria [17]. All control and a subset of 230 PSP (series 1) cases were genotyped in our prior study [8]. All LBD-NP and an additional 810 PSP cases (series 2) were genotyped in this study

*PSP* progressive supranuclear palsy, *LBD-NP* Lewy body disease, neuropathologic diagnosis

The Mayo Clinic Institutional Review Board (IRB) approved all procedures for this study and appropriate protocols were followed. All participants or next-of-kin were properly consented for this study.

### Genotyping and Sequencing

All control and a subset of 230 PSP (PSP Series 1) participants had genotypes obtained in our prior study [8]. We genotyped all 973 LBD-NP and an additional 810 PSP (PSP Series 2) patients using the same methods.

DNA was extracted from blood using AutoGenFlexStar (AutoGen) and FlexiGene Chemistry (Qiagen) or brain using AutoGen 245T using standard protocols. Genotyping was performed using TaqMan assays (ABI3_rs616338, C_2270073_20; PLCG2_rs72824905, C_97909430_10) following manufacturers’ protocol, using a QuantStudio 7 Flex Detection System with a 384-Well Block Module (Applied Biosystems, Foster City, CA).

All minor allele carriers (*ABI3_*rs616338-T, *PLCG2*_rs72824905-G) were confirmed using Sanger Sequencing. Polymerase chain reaction (PCR) primers with the following sequences were used to amplify and sequence the genomic region flanking the mutations: *ABI3* 5′-CTTCCTGCTCGCACCCGAC-3′, 5′-CTAATGCAGCATCCCCAACT-207 3′, *PLCG2* 5′- CCATAAATGAGGGCTCTCAG-3′, 5′-CATACCCACCTCACCCTTGT-3′. PCR products were purified using the Agencourt AMPure protocol (Beckman Coulter, CA) and sequenced using a Big Dye Terminator v3.1 Cycle Sequencing Kit (Applied Biosystems). Sequencing reactions were purified using Agencourt CleanSEQ (Beckman Coulter, CA) and run on an ABI33730xl Genetic Analyzer (Applied Biosystems). Sequences were analyzed using Sequencher 4.8 (Gene Codes Corporation, MI).

### Neuropathology phenotypes

We tested the association of *ABI3*_rs616338 and *PLCG2*_rs72824905 variants with neuropathology traits. We used the following neuropathology traits available on the autopsied patients in the genetic association analyses:

Braak scores [6, 7] were available for 971 LBD-NP and 1039 PSP patients. Thal phase [23] was available for 768 LBD-NP and 1020 PSP participants.

In addition, 841 PSP patients had continuous quantitative neuropathology measures for four distinct tau lesions, namely neurofibrillary tangles (NFT), oligodendroglial coiled bodies (CB), tufted astrocytes (TA), and tau neuropil threads (TAUTH). These patients also had the combined burden of tau neuropathology (overall) as an additional neuropathology trait for analysis. We utilized these PSP neuropathology traits as previously described in detail [3].

All neuropathology phenotypes are described in detail in Additional file 1: This file includes detailed methods on the acquisition of the neuropathology phenotypes.

### Statistical analyses

#### Disease risk association

We tested the association of *ABI3_*rs616338-T and *PLCG2*_rs72824905-G with disease risk in neuropathologically diagnosed LBD-NP and PSP cohorts, using logistic regression (LR) and Fisher’s exact test, implemented in PLINK [20]. An additive model was applied for the SNP minor allele. Each disease cohort was compared with the control participants previously described [8]. All disease and control participants had complete age and sex information and were older than 60 years of age at time of last evaluation. Age is defined as age of death for all autopsy participants, both patients and controls. Age at last clinical evaluation is utilized as the age for clinically normal controls (Table 1). We adjusted for sex and age in all analyses. We also adjusted for *APOE* ε4 dose in the LBD-NP analyses, given prior reported association of this variant with LBD-NP [9].

#### Neuropathology association

We tested the association of *ABI3_*rs616338-T and *PLCG2*_rs72824905-G with the neuropathology phenotypes. Multivariable ordinal regression was performed in R for Braak stage and Thal phase using the rms package. Age and sex were used as covariates in all association tests. For statistical analyses, the ordinal variable for Braak scores was encoded as 13 levels (0–6 in increments of 0.5) and the ordinal variable for Thal phase was encoded as 6 levels (0–5). Analyses were conducted for PSP and LBD-NP patients separately, as well as combined.

Continuous quantitative tau measures from PSP participants were also tested for their associations with the genetic variants, using multivariable linear regression analysis in PLINK [20]. We applied an additive model for SNP minor allele and adjusted for age and sex as covariates. Box plots for SNP associations with neuropathology latent traits were generated in R, as previously described [4].

### Power analysis

Power was estimated by running simulations of data according to each sample size for each variant and recording the observed proportion of results that had *p* value < 0.05. We estimated power with 1600 simulations in each case to ensure adequate precision, as this would allow us to estimate power as a percentage with standard error of 0.1% or less. Any estimates of power that were between 75 and 85 were re-estimated with 6400 simulations. To ensure that we did not overstate power, we rounded all estimates down to the nearest whole percent. Power estimates were also adjusted so as to adhere to monotonicity relationships. Power estimates are delineated in Additional file 2: Table S1.

## Results

### Association of *ABI3_rs616338* and *PLCG2_rs72824905* variants with risk of neuropathologically diagnosed LBD-NP and PSP

In this study, we tested the association of *ABI3*_rs616338-T and *PLCG2*_rs72824905-G with disease risk in 973 LBD-NP and 1040 PSP patients all of whom were neuropathologically diagnosed (Table 1). All genotypes maintained Hardy–Weinberg equilibrium in all of the groups tested.

The autopsy-confirmed LBD-NP cohort was comprised of high, intermediate and low categories for likelihood of typical clinical DLB-CL, based on their LRP and concurrent AD neuropathology burden defined by Braak stage [17]. By definition, the high likelihood DLB-CL category has no samples with Braak stages of 5–6 and the intermediate category has no samples with Braak stages < 3. In our cohort, 60% of the high likelihood typical clinical DLB-CL cases had Braak stages 3–4, whereas 62% of the intermediate and 68% of the low likelihood categories had Braak stages of 5–6 (Additional file 2: Table S2). In the low likelihood category, frequencies of cases with Braak 0–2 (18%) and 3–4 (15%) were similar.

In the combined LBD-NP series encompassing all categories, there was no statistically significant association with either *ABI3*_rs616338 or *PLCG2*_rs72824905. *ABI3*_rs616338-T was more frequently observed in cases, but did not achieve statistical significance in either logistic regression (LR) (OR = 1.27, 95% CI 0.79–2.06, *p* = 0.329) or Fisher’s exact test (Fisher’s) (OR = 1.33, 95% CI 0.84–2.09, *p* = 0.216) (Table 2). When the sub-categories of LBD-NP series were analyzed separately, the intermediate category achieved statistically significant disease risk association with *ABI3*_rs616338-T both with LR (OR = 2.65, 95% CI 1.46–4.83, *p* = 0.001) and Fisher’s (OR = 2.63, 95% CI 1.49–4.63, *p* = 0.003) tests. *PLCG2*_rs72824905-G was more frequently observed in 3346 controls (MAF_Control_ = 0.010) than in the combined LBD-NP series (MAF_DLB_ = 0.006) and its sub-categories, but this was not statistically significant (Table 2).

**Table 2:** Disease associations of autopsy-confirmed LBD-NP and PSP patients

SNP	Disease	Cohort	N		Genotype counts		MAF		Logistic regression			Fisher’s exact test		
			Cases	Controls	Cases	Controls	Cases	Controls	*p* value	OR	95% CI	*p* value	OR	95% CI
*ABI3* rs616338	LBD-NP	Low	262	3351	0/6/256	0/68/3283	0.011	0.010	0.850	1.09	0.46–2.58	0.656	1.13	0.49–2.62
		Intermediate	286	3351	0/15/271	0/68/3283	0.026	0.010	**0.001**	2.65	1.46–4.83	**0.003**	2.63	1.49–4.63
		High	421	3351	0/5/416	0/68/3283	0.006	0.010	0.165	0.51	0.2–1.31	0.347	0.58	0.23–1.45
		Combined LBD-NP	969	3351	0/26/943	0/68/3283	0.013	0.010	0.329	1.27	0.79–2.06	0.216	1.33	0.84–2.09
	PSP	PSP Series 1	230	3351	0/4/226	0/68/3283	0.009	0.010	0.729	0.83	0.3–2.35	1.000	0.86	0.31–2.36
		PSP Series 2	808	3351	0/20/788	0/68/3283	0.012	0.010	0.284	1.33	0.79–2.25	0.418	1.22	0.74–2.02
		Combined PSP	1038	3351	0/24/1014	0/68/3283	0.012	0.010	0.359	1.26	0.77–2.06	0.622	1.14	0.71–1.82
*PLCG2* rs72824905	LBD-NP	Low	262	3346	0/2/260	0/67/3279	0.004	0.010	0.152	0.35	0.08–1.47	0.239	0.38	0.09–1.55
		Intermediate	286	3346	0/5/281	0/67/3279	0.009	0.010	0.567	0.76	0.29–1.97	1.000	0.87	0.35–2.17
		High	420	3346	0/5/415	0/67/3279	0.006	0.010	0.320	0.62	0.25–1.58	0.345	0.59	0.24–1.47
		Combined LBD-NP	968	3346	0/12/956	0/67/3279	0.006	0.010	0.107	0.59	0.31–1.12	0.136	0.62	0.33–1.14
	PSP	PSP Series 1	230	3346	0/8/222	0/67/3279	0.017	0.010	0.238	1.60	0.73–3.47	0.149	1.75	0.84–3.67
		PSP Series 2	809	3346	0/12/797	0/67/3279	0.007	0.010	0.395	0.76	0.4–1.44	0.393	0.74	0.40–1.37
		Combined PSP	1039	3346	0/20/1019	0/67/3279	0.010	0.010	0.741	0.91	0.54–1.56	1.000	0.96	0.58–1.59

Nominally significant *p* values < 0.05 are shown in bold

Results of multivariable logistic regression and Fisher’s exact test analysis using the additive model for association of *ABI3*_rs616338-T and *PLCG2*_rs72824905-G with LBD-NP and PSP disease status. The following covariates were applied: LBD-NP: sex, age, and *APOE* ε4 dosage. PSP: sex and age

Genotypes for ABI3(rs616338)-(TT/CT/CC) and PLCG2(rs72824905)-(GG/CG/CC)

*PSP* progressive supranuclear palsy, *LBD-NP* Lewy body disease, neuropathologic diagnosis, *MAF* minor allele frequency, *OR* odds ratio, *CI* confidence interval

In this study, our total autopsy-confirmed PSP series had 1039 participants, comprised of the previously genotyped group (Series 1, n = 230) [8] and the 810 newly genotyped PSP patients (Series 2). Neither *ABI3*_rs616338 nor *PLCG2*_rs72824905 had statistically significant association with PSP risk in the combined group or each series alone (Table 2). In PSP patients from the smaller Series 1, we had previously identified OR estimates for these variants that had opposite trends to that for AD [8]. In our current study, these opposite trends are no longer observed in the much larger Series 2 PSP patients or the combined PSP series, where neither variant has significant PSP risk association.

### Association of *ABI3_rs616338* and *PLCG2_rs72824905* variants with neuropathologic phenotypes

In this study we leveraged the existing data from neuropathologically diagnosed LBD-NP and PSP patients to determine the influence of the *ABI3*_rs616338 and *PLCG2*_rs72824905 variants on several neuropathologic features. Braak stage and Thal phase measures were available in both PSP and LBD-NP cohorts. Additionally, continuous quantitative neuropathology measures for four tau lesions [1, 3] were analyzed in 841 PSP patients.

#### Association of *ABI3_rs616338* and *PLCG2_rs72824905* variants with Braak stage and Thal phase

Categorical neuropathology measures for tau and amyloid ß (Aß) were available for a total of 2008 autopsied patients as Braak stage [6, 7] and for 1246 patients with Thal phase [23], respectively. Patients with Braak stage information were comprised of 1037 PSP and 971 LBD-NP and those for Thal phase had 1020 PSP and 768 LBD-NP cases. We tested for associations between the genetic variants and these two neuropathologic measures in both the combined neurodegenerative disease groups and in each diagnostic group individually (Table 3). Older age and female sex were associated with greater Aß and tau neuropathology. All analyses were appropriately adjusted for age and sex.

**Table 3:** Braak and Thal associations in LBD-NP and PSP patients

	Variable	PSP and LBD-NP Combined					PSP					LBD-NP				
		BETA	SE	L95	U95	*p*	BETA	SE	L95	U95	*p*	BETA	SE	L95	U95	*p*
Thal	*ABI3_*rs616338_T	0.293	0.268	− 0.233	0.818	0.275	0.287	0.360	− 0.418	0.993	0.425	0.464	0.422	− 0.364	1.292	0.272
	Age	0.053	0.006	0.042	0.064	**< 1E−07**	0.044	0.008	0.028	0.060	**5.43E−08**	0.023	0.008	0.008	0.038	**0.002**
	Female Sex	0.250	0.086	0.082	0.418	**0.004**	0.335	0.117	0.106	0.563	**0.004**	0.614	0.138	0.343	0.884	**8.74E−06**
	*PLCG2_*rs72824905_G	− 0.101	0.357	− 0.802	0.599	0.777	0.010	0.438	− 0.849	0.870	0.981	0.652	0.733	− 0.785	2.090	0.374
	Age	0.053	0.006	0.042	0.064	**< 1E−07**	0.045	0.008	0.029	0.061	**1.93E−08**	0.023	0.008	0.008	0.038	**0.003**
	Female Sex	0.245	0.086	0.077	0.414	**0.004**	0.326	0.117	0.098	0.555	**0.005**	0.620	0.138	0.350	0.891	**7.10E−06**
Braak	*ABI3_*rs616338_T	0.070	0.255	− 0.429	0.569	0.784	− 0.08	0.348	− 0.762	0.603	0.819	0.253	0.367	− 0.466	0.973	0.490
	Age	0.074	0.005	0.064	0.084	**< 1E−07**	0.106	0.008	0.090	0.122	**< 1E−07**	0.011	0.006	− 0.001	0.024	0.065
	Female Sex	0.210	0.079	0.054	0.366	**0.008**	0.254	0.111	0.036	0.473	**0.023**	0.594	0.118	0.364	0.825	**4.42E−07**
	*PLCG2_*rs72824905_G	− 0.822	0.315	− 1.439	− 0.204	**0.009**	− 0.996	0.397	− 1.773	− 0.218	**0.012**	− 0.292	0.505	− 1.283	0.698	0.563
	Age	0.074	0.005	0.065	0.084	**< 1E−07**	0.105	0.008	0.090	0.121	**< 1E−07**	0.011	0.006	− 0.001	0.024	0.064
	Female Sex	0.199	0.079	0.043	0.355	**0.012**	0.249	0.111	0.031	0.467	**0.025**	0.588	0.118	0.358	0.819	**5.88E−07**

Nominally significant *p* values < 0.05 are shown in bold

Results of multivariable ordinal regression using the additive model for association of *ABI3*_rs616338-T and *PLCG2*_rs72824905-G with Braak and Thal in patients with PSP, LBD-NP and combined PSP + LBD-NP. Analyses were adjusted for age and sex

*SE* standard error, *L95* lower 95% limit, *U95* upper 95% limit

In the combined neurodegenerative disease group of autopsied PSP and LBD-NP patients, *PLCG2*_72824905-G was associated with lower Braak stage (ß = − 0.822, 95% CI − 1.439 to − 0.204, *p* = 0.009) (Table 3). When the disease groups were analyzed separately, *PLCG2*_72824905-G had negative effect size estimates in both PSP and LBD-NP groups, aligned with a suppressive effect of this variant on tau pathology. This association was significant in the PSP patients (ß = − 0.995, 95% CI − 1.773 to − 0.218, *p* = 0.012), but not in LBD-NP (ß = − 0.292, 95% CI − 1.283 to 0.698, *p* = 0.563). None of the other genetic associations were significant.

#### Association of ABI3_rs616338 and PLCG2_rs72824905 variants with quantitative tau neuropathology

Quantitative tau neuropathology measures were available for four characteristic tau lesions observed in PSP brains, namely neurofibrillary tangles (NFT), coiled bodies (CB), tufted astrocytes (TA) and tau threads (TAUTH), present in neurons, oligodendrocytes, astrocytes, and white matter, respectively [10]. 841 PSP patients had continuous quantitative measures for these four tau neuropathologies and a measure of overall tau burden generated from semi-quantitative tau pathology counts from 19 brain regions [1, 3]. To identify *ABI3*_rs616338-T and *PLCG2*_72824905-G associations with tau neuropathology in PSP we performed a multivariable linear regression analysis.

**Fig. 1:**
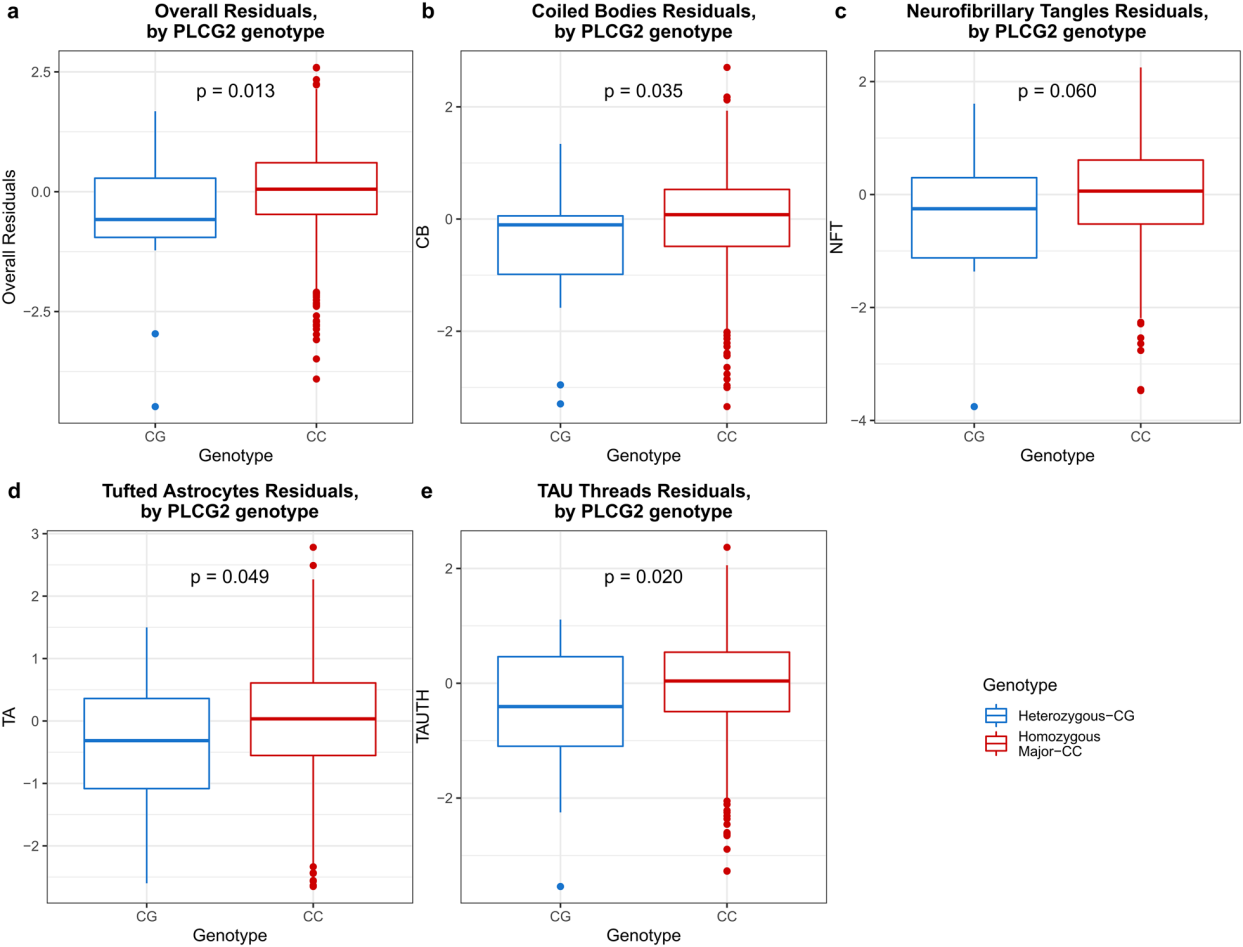
Box plots for *PLCG2*_rs72824905 associations with quantitative tau neuropathology in PSP. Box plots of quantitative tau neuropathology measures for **a** overall, **b** coiled body (CB), **c** neurofibrillary tangle (NFT), **d** tufted astrocyte (TA), and **e** tau thread (TAUTH) measures by *PLCG2*_rs72824905 genotype. Blue indicates distribution of gene expression residuals for heterozygotes (CG); red indicates the same for major homozygotes (CC). Quantitative tau measures were adjusted for age and sex and residuals were plotted

Carriers of the *ABI3*_rs616338-T variant showed a trend for greater NFT burden in PSP (ß = 0.38, 95% CI − 0.022 to 0.786, *p* = 0.064) (Table 4) but there were no associations or trends for CB, TA, TAUTH, or Overall tau burden for this variant. Importantly, carriers of *PLCG2*_72824905-G showed association with reduced tau pathology for all lesions with the following effect size estimates: CB (ß = − 0.487, 95% CI − 0.938 to − 0.036, *p* = 0.035), NFT (ß = − 0.449, 95% CI − 0.916 to 0.018, *p* = 0.060), TA (ß = − 0.482, 95% CI − 0.96 to − 0.004, *p* = 0.049), TAUTH (ß = − 0.546, 95% CI − 1.006 to − 0.085, *p* = 0.020), and Overall (ß = − 0.638, 95% CI − 1.139 to − 0.136, *p* = 0.013) (Table 4). Visualization of these associations by box plots demonstrates the reduced levels of tau measures in the carriers of *PLCG2*_72824905-G, for all tau lesions (Fig. 1). These findings suggest that *PLCG2*_72824905-G may have a role in reducing or limiting tau neuropathology.

**Table 4:** Quantitative tau neuropathology trait associations in PSP

SNP	Tau traits tested	N	Genotype counts	BETA	*p*	95% CI
*ABI3* rs616338	CB	840	0/19/821	0.057	0.773	− 0.333 to 0.448
	NFT	840	0/19/821	0.382	0.064	− 0.022 to 0.786
	TA	840	0/19/821	− 0.096	0.649	− 0.511 to 0.318
	TAUTH	840	0/19/821	0.204	0.317	− 0.195 to 0.603
	Overall	840	0/19/821	0.187	0.400	− 0.248 to 0.622
*PLCG2* rs72824905	CB	841	0/14/827	− 0.487	**0.035**	− 0.938 to − 0.036
	NFT	841	0/14/827	− 0.449	0.060	− 0.916 to 0.018
	TA	841	0/14/827	− 0.482	**0.049**	− 0.96 to − 0.004
	TAUTH	841	0/14/827	− 0.546	**0.020**	− 1.006 to − 0.085
	Overall	841	0/14/827	− 0.638	**0.013**	− 1.139 to − 0.136

Nominally significant *p* values < 0.05 are shown in bold

Results of multivariable linear regression using the additive model for association of *ABI3*_rs616338-T and *PLCG2*_rs72824905-G with neuropathologic traits in autopsied PSP subjects. All analyses adjust for sex and age

*CI* confidence interval. Genotypes for ABI3(rs616338)-(TT/CT/CC) and PLCG2(rs72824905)-(GG/CG/CC). Neuropathology traits: *CB* oligodendroglial coiled bodies, *NFT* neurofibrillary tangles, *TA* tufted astrocytes, *TAUTH* Tau neurophil threads

## Discussion

In this study we evaluated the association of the missense AD risk variants *ABI3*_rs616338-T and *PLCG2*_rs72824905-G with both disease risk and neuropathology in autopsy-confirmed cohorts comprised of 973 LBD-NP and 1040 PSP patients. To our knowledge, this is one of the largest studies to date of these variants in these purely neuropathologically diagnosed neurodegenerative diseases. Our findings suggest that the protective role of *PLCG2*_rs72824905-G may be through a suppressive effect on tau pathology. We also find evidence of *ABI3*_rs616338-T association with LBD-NP risk in a sub-category of patients with diffuse neocortical or limbic LB, concurrently with moderate or high AD neuropathology, respectively. Finally, in our sizable autopsy-confirmed PSP cohort, we find no evidence of association with disease risk for either variant.

In our previous study [8], we analyzed both variants in 306 DLB patients of whom 22% were autopsy-confirmed (LBD-NP) and 231 neuropathologically diagnosed PSP patients, in addition to AD, PD and MSA cohorts. The current study achieved a > 3 and > 4.5 fold increase in sample size for LBD-NP and PSP, respectively, compared to our prior work [8].

Our lack of statistically significant disease risk association with *PLCG2*_rs72824905-G and our autopsy-confirmed PSP cohort is consistent with the findings from the multi-center van der Lee et al. [24] study, which analyzed 882 PSP patients of whom 29% were autopsy-confirmed. In a GWAS of 2165 PSP cases (around 58% autopsy-confirmed), no associations were identified at the *PLCG2* or *ABI3* loci [13], similar to our results in this study.

Likewise, we did not find statistically significant associations with either variant and disease risk in our combined autopsy-confirmed cohort of 973 LBD-NP patients. This is in contrast to the multi-center van der Lee et al. [24] study, which detected association between *PLCG2*_rs72824905-G and reduced disease risk in 1446 DLB patients, of whom 11% had neuropathologic diagnosis. In a GWAS of 1743 DLB patients, of whom 1324 were autopsy-confirmed [11], no associations were identified at the *PLCG2* or *ABI3* loci, although significant associations were reported for *APOE*, *SNCA* and *GBA*. More recently, whole exome sequencing of 1118 autopsy-confirmed DLB patients [19] did not identify significant association with *PLCG2*_rs72824905-G.

These collective results suggest either that variants in *ABI3* and *PLCG2* do not play as significant a role in PSP and LBD-NP as in AD or that the existing studies are underpowered. Indeed, the initial report of AD risk associations with these rare variants was based on 37,022 AD cases and 48,402 controls [22], which is at least an order of magnitude greater than all studies combined for PSP or LBD-NP. Future efforts should be placed on combining data from the existing studies as well as expanding autopsy-confirmed cohorts of these other neurodegenerative diseases.

Even though our study lacked power to detect disease risk association for these variants, we nonetheless leveraged the available in-depth neuropathology data to investigate their potential impact on specific neuropathologies. We first evaluated sub-categories of LBD-NP to determine whether any associations were driven by concurrent AD neuropathology, utilizing established criteria [17]. We did not find statistically significant evidence for association of *PLCG2*_rs72824905-G with any of the three DLB sub-categories. In contrast, *ABI3*_rs616338-T was associated with increased risk of LBD-NP only in the intermediate category, which remained significant after adjusting for multiple testing. These findings may suggest that *ABI3*_rs616338-T associates with AD, but not LBD-NP, as the intermediate category has a higher burden of AD pathology unlike the high category. Alternatively, this variant may drive a specific combination of Lewy-related and AD pathology.

Our study also aimed to delineate the roles of these missense variants in influencing Aß and/or tau neuropathology as defined by Thal phase [23] or Braak stage [7], respectively. There was no association between *ABI3*_rs616338-T and either pathology. *PLCG2*_rs72824905-G associated with lower Braak scores and lower quantitative tau neuropathology, which is congruent with its protective effect seen in AD [8, 22, 24], some studies of DLB-CL [8, 24] and frontotemporal dementia (FTD) [24]. Tau neuropathology is *sine qua non* in AD, frequently concurrent in LBD [5] and the main pathology in frontotemporal lobar degeneration due to tau (FTLD-tau), which constitutes about 40% of all FTLD [18]. As tau is a common pathology in these conditions, it is plausible that a genetic variant that reduces risk across these diseases acts via suppressing tau neuropathology.

It is notable that the tau-suppressive effect of *PLCG2*_rs72824905-G is more pronounced in PSP Braak stage phenotype (beta = − 0.995, *p* = 0.01) than that for LBD (beta = − 0.292, *p* = 0.6). Braak scores are on average higher in our 971 LBD (mean = 3.9) than in 1036 PSP patients (mean = 2.5). This suggests that *PLCG2*_rs72824905-G may have a stronger suppressive effect on tau in earlier Braak stages.

Our results are aligned with those from a recent study of longitudinally followed clinical patients with mild cognitive impairment (MCI), where *PLCG2*_rs72824905-G was associated with lower cerebrospinal fluid (CSF) levels of pTau181 and cognitive decline [15]. Collectively, our study which is focused on tau neuropathology and the published work on CSF tau [15] are consistent with a model where the effect of *PLCG2*_rs72824905-G in suppressing hyperphosphorylated tau may be most pronounced in the early stages of tau cortical deposition.

In addition to a suppressive effect as detected by Braak scores, there was also significant association of *PLCG2*_rs72824905-G and reduced quantitative neuropathology in PSP for different tau lesions, namely oligodendroglial coiled bodies (CB), tufted astrocytes (TA), tau threads (TAUTH) and neurofibrillary tangles (NFT). Despite a consistent effect on tau neuropathology in PSP, *PLCG2*_rs72824905-G does not have any effect on risk of this neurodegenerative disease. There may be several explanations for these results. Unlike AD, which has multiple neuropathologies, PSP is a primary tauopathy. It is possible that suppression of tau neuropathology by *PLCG2*_rs72824905-G may have a protective effect in neurodegeneration in the context of other neuropathologies but not when tau is the primary proteinopathy. Consistent with this possibility is the the suppressive effect of *PLCG2*_rs72824905-G on CSF pTau181, which was most pronounced in those MCI patients who also had evidence of Aß deposits based on low CSF Aß42 levels [15].

Collectively, these results may support the hypothesis that Aß and possibly also α-synuclein amplify the tau-suppressive effects of *PLCG2*_rs72824905-G in the early stages of tau cortical deposition. Using our human brain gene expression data [2, 3], we previously showed that *PLCG2* resides in a brain co-expression network enriched for microglial genes and that brain *PLCG2* levels were higher in AD but not in PSP compared to controls [8]. We also discovered higher brain levels of *plcg2* in two mouse models of amyloidosis compared to non-transgenic littermates [22]. Recently, the protective *PLCG2*_rs72824905-G variant was shown to be a functional hypermorph, which increased the enzymatic activity of PLCγ2 [16]. Our human and mouse gene expression results suggest that Aß but not tau leads to increased brain *PLCG2* levels, either through microgliosis, microglial activation or both. Hence, the combination of increased levels of brain PLCG2, due to presence of Aß and higher enzymatic activity imposed by *PLCG2*_rs72824905-G may be necessary for an ultimately protective effect on disease risk. Though supported by multiple lines of evidence, this hypothesis requires further confirmation by testing the effects of *PLCG2*_rs72824905-G on neuropathology and other outcomes in model systems of different proteinopathies. To determine whether α-synuclein, like Aß, also leads to elevated brain levels of *PLCG2*, LBD cohorts without AD neuropathology need to be evaluated in transcriptome studies. Additionally, the influence of *PLCG2*_rs72824905-G on tau neuropathology and disease risk needs exploration in other conditions such as corticobasal degeneration (CBD) and FTLD-tau to determine the generalization of our results to other primary tauopathies.

Our study has numerous strengths, including assessment of two missense AD risk variants in two large autopsy-confirmed LBD-NP and PSP cohorts, investigating their effects on LBD-NP sub-categories representing different levels of LB and AD pathologies, detailed analyses of Aß and tau as endophenotypes including quantitative tau neuropathologies in 841 PSP patients. Despite these strengths, our study has some shortcomings. Even with our sizeable autopsy-confirmed cohorts, we were underpowered to detect associations given the low frequency of these variants. This raises the possibility of both false negative and false positive findings, although we note that the *ABI3*_rs616338-T association in the intermediate category would withstand correction for multiple testing. While we were able to assess both LBD-NP and PSP cohorts for genetic associations with Braak stage and Thal phase, quantitative tau measures were available only for the latter. Our study was conducted in self-reported Caucasian participants, which may not be a true representation of their genetic ancestry. Additionally, these results cannot be generalized to non-Caucasian populations. We determined previously that *ABI3*_rs616338-T and *PLCG2*_rs72824905-G are even rarer in African Americans than in Caucasians [8], suggesting that they have a smaller or no effect on AD risk in African Americans. Future studies should screen *ABI3* and *PLCG2* in sizable non-Caucasian cohorts to uncover the spectrum of genetic variants in these genes that may influence disease risk and neuropathology in non-Caucasian populations. Finally, both disease and neuropathologic associations should be investigated in other autopsy-confirmed neurodegenerative diseases to establish the generalizability of our conclusions to other conditions.

In summary, in our study of 973 LBD-NP and 1040 PSP autopsy-confirmed patients, we find evidence that the protective effect of *PLCG2*_rs72824905-G may be driven by suppressing tau especially in the earlier stages of its cortical deposition and may require presence of another proteinopathy, such as Aß to confer reduced disease risk. There is evidence of increased risk with *ABI3*_rs616338-T in a subset of LBD-NP patients with moderate to high AD pathology. These findings highlight potential mechanisms of action for these variants and exemplify utilization of detailed neuropathology phenotypes to untangle precise effects of genetic factors in these complex and heterogeneous neurodegenerative diseases.

## Supplementary information

**Additional file 1: Supplementary Text** This file includes detailed methods on the acquisition of the neuropathology phenotypes

**Additional file 2: Table S1a**. Power at 5% significance level by odds ratio for 3351 controls and specified number of cases, for *ABI3* with MAF of 0.8 % in controls. **Table S1b** Power at 5% significance level by odds ratio for 3351 controls and specified number of cases, for *PLCG2* with MAF of 1.09 % in controls. **Table S2**. (A) Braak stage and (B) Thal phase distribution in the PSP and LBD cohorts based on (1) *ABI3*_rs616338_C_T and (2) *PLCG2*_rs72824905_C_G genotypes. The total number of subjects and percentage (N (%)) are shown for those subjects with Braak and Thal measures. For each series, the number of carriers with the minor allele, the major allele, and all subjects with a genotype for the given SNP are shown, respectively. The percentage is the N for each neuropathologic category for a given genotype divided by the total N of each series. Braak stage and Thal phase categories are defined as per prior published criteria. Abbreviations: *PSP* progressive supranuclear palsy, *LBD* dementia with Lewy bodies. Genotypes for *ABI3*(rs616338)-(TT/CT/CC) and *PLCG2*(rs72824905)-(GG/CG/CC). Given their rarity, there were no individuals with *ABI3*(rs616338)_TT or *PLCG2*(rs72824905)_GG genotypes.

## Data Availability

All summary results are provided within the manuscript.

## Abbreviations

Aß: amyloid ß
AD: Alzheimer’s disease
CB: oligodendroglial coiled bodies
CSF: cerebrospinal fluid
DLB-CL: dementia with Lewy bodies, diagnosed clinically
FTD: frontotemporal dementia
FTLD-tau: frontotemporal lobar degeneration with tau pathology
LB: Lewy bodies
LBD-NP: Lewy body disease, diagnosed neuropathologically
LR: logistic regression
LRP: Lewy-related pathology
MAF: minor allele frequency
MCI: mild cognitive impairment
NFT: neurofibrillary tangle
OR: odds ratio
PSP: progressive supranuclear palsy
TA: tufted astrocytes
TAUTH: tau neuropil threads
