# Supplementary material for: Association of ABI3 and PLCG2 missense variants with disease risk and neuropathology in Lewy body disease and progressive supranuclear palsy
Source: Acta Neuropathol Commun. 2020 Oct 22;8:172. doi: 10.1186/s40478-020-01050-0 (PMC7579984; doi:10.1186/s40478-020-01050-0)
Supplement: Supplementary file 1 — Additional file 1: Supplementary Text This file includes detailed methods on the acquisition of the neuropathology phenotypes [file 40478_2020_1050_MOESM1_ESM.docx]

**Association of *ABI3* and *PLCG2* missense variants with disease risk and neuropathology in Lewy body disease and progressive supranuclear palsy**

Samantha L. Strickland^1*^, Helene Morel^1*^, Christian Prusinski^1^, Mariet Allen^1^, Tulsi A. Patel^1^, Minerva M. Carrasquillo^1^, Olivia J. Conway^1^, Sarah J. Lincoln^1^, Joseph S. Reddy^2^, Thuy Nguyen^1^, Kimberly G. Malphrus^1^, Alexandra I. Soto^1^, Ronald L. Walton^1^, Julia E. Crook^2^, Melissa E. Murray^1^, Bradley F. Boeve^3^, Ronald C. Petersen^3^, John A. Lucas^4^, Tanis J. Ferman^4^, Ryan J. Uitti^5^, Zbigniew K. Wszolek^5^, Owen A. Ross^1^, Neill R. Graff-Radford^5^, Dennis W. Dickson^1^, Nilüfer Ertekin-Taner^1,5#^

^1^Department of Neuroscience, Mayo Clinic Florida, Jacksonville, FL 32224, USA

^2^Department of Health Sciences Research, Mayo Clinic Florida, Jacksonville, FL 32224, USA

^3^Department of Neurology, Mayo Clinic Minnesota, Rochester, MN 55905, USA

^4^Department of Psychiatry and Psychology, Mayo Clinic Florida, Jacksonville, FL 32224, USA

^5^Department of Neurology, Mayo Clinic Florida, Jacksonville, FL 32224, USA

*: Contributed equally #: Corresponding Author

**Corresponding Author Contact Information:** Mayo Clinic, Departments of Neurology and Neuroscience, 4500 San Pablo Road, Birdsall 3, Jacksonville, FL 32224.

E-mail: taner.nilufer@mayo.edu, Phone: 904-953-7103, FAX: 904-953-7353.

**Supplementary Methods:**

**Neuropathology phenotypes**

For 841 autopsied PSP participants, continuous quantitative neuropathology measures (latent traits) were available for the following tau lesions: neurofibrillary tangles (NFT), oligodendroglial coiled bodies (CB), tufted astrocytes (TA), and tau neuropil threads (TAUTH), and the combined burden of neuropathology (overall). The generation of these latent traits has been previously described in detail[1]. Briefly, semi-quantitative counts were generated by a single neuropathologist (DWD) using CP13 immunostained sections from 19 brain regions affected in PSP, which include: basal nucleus, caudate/ putamen, globus pallidus, hypothalamus, motor cortex, subthalamic nucleus, thalamic fasciculus, ventral thalamus, cerebellar white matter, dentate nucleus, inferior olive, locus coeruleus, medullary tegmentum, midbrain tectum, oculomotor complex, pontine base, pontine tegmentum, red nucleus, and substantia nigra. Counts across all 19 brain regions were used to estimate neuropathological latent traits, using the R statistical software “ltm” package[5] (URL: <http://www.jstatsoft.org/v17/i05/>).

The neuropathology measures were each on a 0 to 3 scale and were used to create continuous scores for the degree of pathology, for each of the four pathological lesions (NFT, CB, TA, TAUTH), based on a latent trait approach[2,5]. An overall latent variable was also calculated by using the semi-quantitative scores for all four lesion types in all regions. These scores are an estimate of an assumed underlying level of pathology severity that all individual scores are dependent on, or correlated with.

Braak stage and Thal phase were also determined by a single neuropathologist (DWD). Braak stage is based on the whether the bulk of the abnormal tau protein is detectable in the transentorhinal and entorhinal regions (stages 1-2), in the limbic allocortex and adjoining neocortex (stages 3-4), or in the neocortex, including the secondary and primary fields (stages 5-6)[3,4]. Thal phase is based on the characterization of amyloid β protein (Aβ) deposits. Phase 1 – neocortex; Phase 2 - additional involvement of allocortical brain regions; Phase 3 – include diencephalic nuclei, the striatum, and the cholinergic nuclei of the basal forebrain; Phase 4 – include brainstem nuclei; Phase 5 is characterized by cerebellar Aβ-deposition[6].

**References:**

1. Allen M, Burgess JD, Ballard T, Serie D, Wang X, Younkin CS, Sun Z, Kouri N, Baheti S, Wang C, Carrasquillo MM, Nguyen T, Lincoln S, Malphrus K, Murray M, Golde TE, Price ND, Younkin SG, Schellenberg GD, Asmann Y, Ordog T, Crook J, Dickson D, Ertekin-Taner N (2016) Gene expression, methylation and neuropathology correlations at progressive supranuclear palsy risk loci. Acta Neuropathol 132:197-211. doi:10.1007/s00401-016-1576-7

2. Bollen KA (2002) Latent variables in psychology and the social sciences. Annu Rev Psychol 53:605-634. doi:10.1146/annurev.psych.53.100901.135239

3. Braak H, Alafuzoff I, Arzberger T, Kretzschmar H, Del Tredici K (2006) Staging of Alzheimer disease-associated neurofibrillary pathology using paraffin sections and immunocytochemistry. Acta Neuropathol 112:389-404. doi:10.1007/s00401-006-0127-z

4. Braak H, Braak E (1991) Neuropathological stageing of Alzheimer-related changes. Acta Neuropathol 82:239-259

5. Rizopoulos D (2006) ltm: An R Package for Latent Variable Modeling and Item Response Theory Analyses. Journal of Statistical Software 17:1-25

6. Thal DR, Rub U, Orantes M, Braak H (2002) Phases of A beta-deposition in the human brain and its relevance for the development of AD. Neurology 58:1791-1800
